# The relationship between lifestyle and negative affect. Executive functioning and emotional regulation as mediators.u

**DOI:** 10.1192/j.eurpsy.2025.691

**Published:** 2025-08-26

**Authors:** I. Ursu, R. S. Balazsi, A. Ursu

**Affiliations:** 1 Psychology, University Babes-Bolyai; 2 Psychiatry, Emergency Hospital of Neurology and Psychiatry, Cluj-Napoca, Romania

## Abstract

**Introduction:**

The increasing prevalence of mental health issues presents a significant challenge for modern societies. There’s a crucial need for quick, affordable interventions that can be widely implemented by practitioners to support as many individuals as possible.

**Objectives:**

In this paper we aimed to investigate the mediating role of executive functions and emotional regulation in the relationship between sleep quality and physical activity, on one hand and negative affect, on the other.

**Methods:**

Our proposed model is a serial mediation model, with executive functions as the first mediator and the two components of emotional regulation as the second mediator. We collected data from 286 participants who completed a series of questionnaires.

**Results:**

The initial model did nit fit the data well. Therefore, we added two paths: the direct relationship between sleep quality and negative affect and the direct relationship between executive functions and negative affect. The final model had a very good fit with the data. Thus, both the hypotheses regarding the direct relationships and those regarding the indirect relationships are supported by the data.

**Image 1:**

**Figure:**
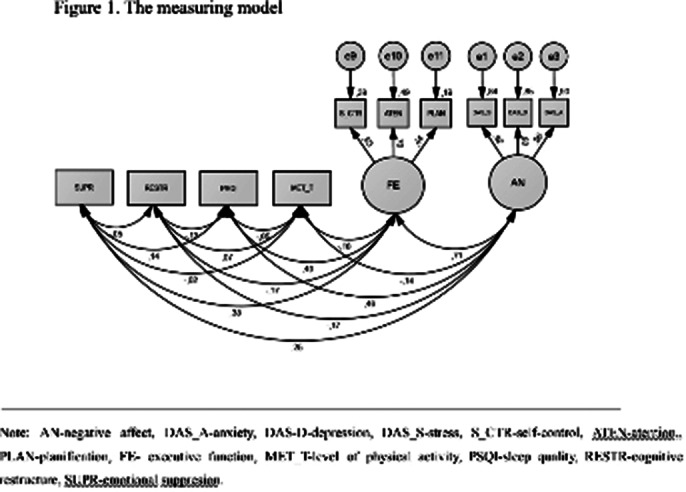

**Image 2:**

**Figure:**
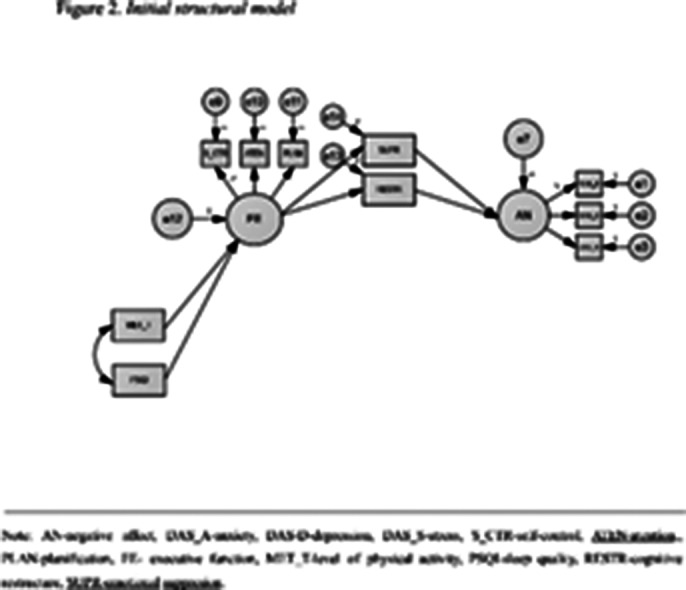

**Image 3:**

**Figure:**
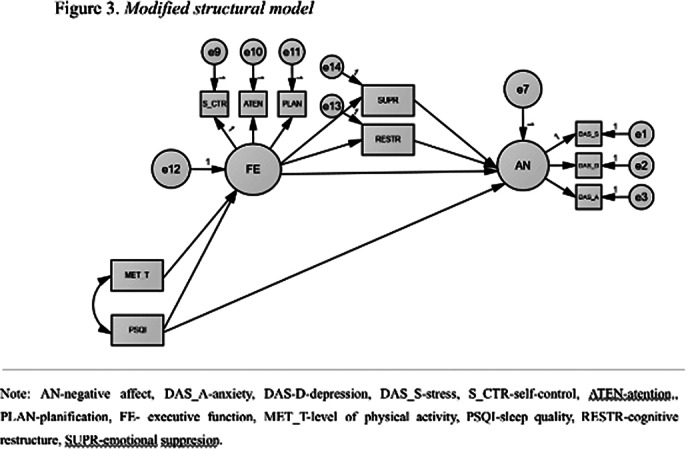

**Conclusions:**

The results highlight the importance of interventions aimed at improving sleep quality and promoting physical activity. These interventions can serve to promote optimal mental health in both clinical and non-clinical populations. Additionally, this research provides a basis for developing effective strategiesfor the prevention and treatment of these populations.

**Disclosure of Interest:**

None Declared

